# Missense Mutations in the *MEFV* Gene Are Associated with Fibromyalgia Syndrome and Correlate with Elevated IL-1β Plasma Levels

**DOI:** 10.1371/journal.pone.0008480

**Published:** 2009-12-30

**Authors:** Jinong Feng, Zhifang Zhang, Wenyan Li, Xiaoming Shen, Wenjia Song, Chunmei Yang, Frances Chang, Jeffrey Longmate, Claudia Marek, R. Paul St. Amand, Theodore G. Krontiris, John E. Shively, Steve S. Sommer

**Affiliations:** 1 Division of Molecular Genetics, Beckman Research Institute, City of Hope, Duarte, California, United States of America; 2 Department of Immunology, Beckman Research Institute, City of Hope, Duarte, California, United States of America; 3 Department of Biostatistics, Beckman Research Institute, City of Hope, Duarte, California, United States of America; 4 Fibromyalgia Treatment Center, Los Angeles, California, United States of America; 5 Department of Molecular Medicine, Beckman Research Institute, City of Hope, Duarte, California, United States of America; New York University, United States of America

## Abstract

**Background:**

Fibromyalgia syndrome (FMS), a common, chronic, widespread musculoskeletal pain disorder found in 2% of the general population and with a preponderance of 85% in females, has both genetic and environmental contributions. Patients and their parents have high plasma levels of the chemokines MCP-1 and eotaxin, providing evidence for both a genetic and an immunological/inflammatory origin for the syndrome (Zhang et al., 2008, Exp. Biol. Med. 233: 1171–1180).

**Methods and Findings:**

In a search for a candidate gene affecting inflammatory pathways, among five screened in our patient samples (100 probands with FMS and their parents), we found 10 rare and one common alleles for *MEFV*, a gene in which various compound heterozygous mutations lead to Familial Mediterranean Fever (FMF). A total of 2.63 megabases of genomic sequence of the *MEFV* gene were scanned by direct sequencing. The collection of rare missense mutations (all heterozygotes and tested in the aggregate) had a significant elevated frequency of transmission to affecteds (*p* = 0.0085, one-sided, exact binomial test). Our data provide evidence that rare missense variants of the *MEFV* gene are, collectively, associated with risk of FMS and are present in a subset of 15% of FMS patients. This subset had, on average, high levels of plasma IL-1β (p = 0.019) compared to FMS patients without rare variants, unaffected family members with or without rare variants, and unrelated controls of unknown genotype. IL-1β is a cytokine associated with the function of the *MEFV* gene and thought to be responsible for its symptoms of fever and muscle aches.

**Conclusions:**

Since misregulation of IL-1β expression has been predicted for patients with mutations in the *MEFV* gene, we conclude that patients heterozygous for rare missense variants of this gene may be predisposed to FMS, possibly triggered by environmental factors.

## Introduction

Fibromyalgia syndrome (FMS) is characterized by chronic, widespread pain in the muscles and joints. FMS is also accompanied by a variety of other common symptoms, including sleep disturbance, fatigue, headache and mood disorders [1]. The prevalence of FMS in the general population is estimated at 2%, where 85% of the affected are females [2]. The current American College of Rheumatology (ACR) criteria for diagnosis/entry into a clinical trial relies on the scoring of 11/18 positive tender points plus widespread pain lasting for more than 3 months [3]. The etiology of FMS is elusive, with proposals ranging from over-sensitivity to pain to chronic infections. Both familial and genetic studies suggest a role for genetic factors in the development of FMS.

Family studies showed a strong familial aggregation of FMS and related conditions [4], [5], [6]. A possible role of genetic and familial factors in this syndrome was studied in 58 offspring originating from 20 complete nuclear families ascertained through affected mothers with FMS [7]. Indeed, a high prevalence of FMS was observed among offspring of FMS mothers. Since psychological and familial factors were not different in children with and without FMS, the high familial occurrence of this syndrome may be attributable to genetic factors [7]. In a more comprehensive family study, Arnold et al. [5] assessed familial aggregation of FMS with measures of tenderness and pain, and familial co-aggregation of FMS with major mood disorder. The odds ratio measuring the odds of FMS in a relative of a proband with FMS versus the odds of FMS in a relative of a proband with rheumatoid arthritis (RA) was 8.5. The number of tender points was significantly higher and the total myalgic score was significantly lower in the relatives of FMS probands compared with relatives of RA probands. The results of this study indicate that FMS and reduced pressure pain thresholds aggregate in families, and that FMS co-aggregates with major mood disorder in families. These findings have clinical and theoretical implications, including the possibility that genetic factors are involved in the etiology of FMS and pain sensitivity.

Studies in search of the genetic predisposition to FMS have been conducted based on the strong evidence of a familial aggregation in FMS. The genes involved in serotoninergic neurotransmission are of special interest in FMS, as this neurotransmitter not only partially mediates central pain perception, but also functions to regulate anxiety related traits often associated with FMS [8], [9], [10], [11], [12]. The silent T102C polymorphism of the 5-HT2A-receptor gene was investigated in 168 FMS patients and 115 healthy controls [13], [14]. A significantly different genotype distribution was shown, in which FMS patients had fewer T/T and more frequent T/C and C/C genotypes as compared to the control population (Fisher's exact test, two-sided, p = 0.008). Genotypes were unrelated to age of onset, duration of disease or psychopathological symptoms. Self-reported information on pain severity was significantly higher in patients of the T/T genotype, which suggested that the T102-allele might be involved in the complex circuits of nociception. The T102C polymorphism might be in linkage disequilibrium with the true functional variant [13].

A second gene investigated, catechol-O-methyltransferase (COMT), encodes an enzyme that inactivates catecholamines and catecholamine-containing drugs. The significance of COMT polymorphisms was assessed in 61 FMS patients and 61 healthy controls [15]. The LL and LH genotypes together were more highly represented in patients than controls (p = 0.024), and HH genotypes in patients were significantly lower than in the control group (p = 0.04). It was concluded that COMT polymorphisms would be of potential pharmacological importance regarding individual differences in the metabolism of catechol drugs and may also be involved in the pathogenesis and treatment of FMS through adrenergic mechanisms, as well as genetic predisposition to FMS [15].

Though several lines of evidence suggest a role for polymorphisms of genes in the serotoninergic, catecholaminergic and dopaminergic [16] systems in the etiopathogenesis of FMS, these polymorphisms are not specific for FMS and are similarly associated with additional co-morbid conditions. Currently, no evidence has emerged to point to a monogenic mode of transmission, while a multi-factorial mode of transmission is generally presumed [17].

Recently, we have investigated the possibility that FMS has an immunological basis [18], an idea that remains controversial [19], [20], [21]. In support of this idea, biopsies of FMS skeletal muscles showed defects that may have been caused (among other possibilities) by an inflammatory disorder [22]. We found that both patients and their parents had high plasma levels of the chemokines MCP-1 (p<0.001) and eotaxin (p<0.01), supporting both the genetic and immunological basis for this syndrome [18]. Possible immunological triggers for FMS include intestinal inflammation such as found in irritable bowel syndrome (IBS), a condition found in a large percentage (ca. 50%) of FMS patients [23], [24].

In a search for possible candidate genes that are associated with conditions such as inflammation of the bowel, as well as other organs, we became interested in the gene *MEFV*, in which a number of mutations cause Familial Mediterranean Fever (FMF). FMF is an autosomal recessive disorder characterized by recurrent attacks of fever and inflammation in the peritoneum, synovium, or pleura, and accompanied by pain [25], [26]. Clinical features show that FMS and FMF have some overlapping symptoms, such as chronic lower body pain, points of tenderness, and widespread pain.

The *MEFV* gene, located on chromosome 6p13.3, has 10 exons and encodes a 781 amino acid protein, termed pyrin. Pyrin contains the PYD domain, which belongs to a member of the six-helix bundle, death-domain superfamily that includes death domains, death effector domains, and caspase activation and recruitment domains (CARDs). The NLR (nucleotide binding domain and leucine rich, or NOD-like receptor, where NOD is an abbreviation for nucleotide oligomerization domain) family of genes also contain the PYD domain [27], [28], [29], [30]. While the pyrin protein is thought to function in apoptotic and inflammatory signaling pathways [31], [32], its exact function has been debated. In a better-studied system, cryopyrin or NLRP3, which also has the PYD domain, is activated to assemble the inflammasome [33], a multi-protein structure that ultimately results in activation of pro-caspase-1 that, in turn, processes pro-IL-1β to IL-1β. Upon release from monocytes, IL-1β causes fever. Notably, rare mutations in the cryopyrin gene (*NLRP3* or *CIAS1*) are dominant, leading to a variety of serious syndromes [34], [35], [36], [37], [38]. The current hypothesis for the role of pyrin in FMF is that wild type pyrin inhibits, while mutated pyrin fails to inhibit, the inflammasome [39]. This situation would be similar to the role of NOD2 in Crohn's disease, in which wild type NOD2, also a member of the NLR family [40], inhibits TLR2 (toll-like receptor-2) activation by peptidoglycan, while mutated NOD2 fails to inhibit TLR2, leading to chronic inflammation in the bowel [41]. More recently, the pyrin-domain-containing protein, HIN-200, has been shown to regulate caspase activation in response to foreign double-stranded DNA [42], further suggesting that members of the NLR gene family, especially the PYD-domain-containing members, regulate inflammation.

To explore the possibility that mutations in the *MEFV* gene may predispose to FMS, we sequenced the regions of likely functional significance in the *MEFV* gene, exons and splice junctions, in 100 FMS probands and their parents (trios), plus a small number of affected siblings. We hypothesized that rare missense variants would, collectively, be found at elevated frequency in affected individuals. This hypothesis is based on the observation that different rare mutations in the *MEFV* gene (<5% population frequency) can lead to FMF when they are compound heterozygous, reports that rare mutations in the related cryopyrin gene act as dominant risk alleles for a variety of syndromes, and published theoretical arguments that low allele frequency can serve as a predictor of functional significance [43]. It is possible that rare mutations on a single haplotype in the *MEFV* gene cause milder symptoms than those in the compound heterozygous state associated with FMF or, more likely, that additional factors present in the environment may trigger FMS.

We found 10 rare missense mutations, occurring in combinations forming 10 distinct haplotypes in 18 families in which we could follow 22 independent transmission events in which a heterozygous parent might have transmitted any of the rare haplotypes to an affected child. We tested our hypothesis by counting the transmissions from heterozygous parents to affected offspring, and found a significant transmission bias (p = 0.0085, exact binomial test, one-sided), indicating a positive association between FMS and rare mutations in the *MEFV* gene. Rare mutations were present in 15 of 100 probands In addition, the subset of FMS patients with rare alleles had elevated plasma levels of IL-1β (p = 0.019). These findings further support the genetic and immunological basis of this syndrome.

## Results

### *MEFV* Variants in FMS Trios

**Figure 1:**
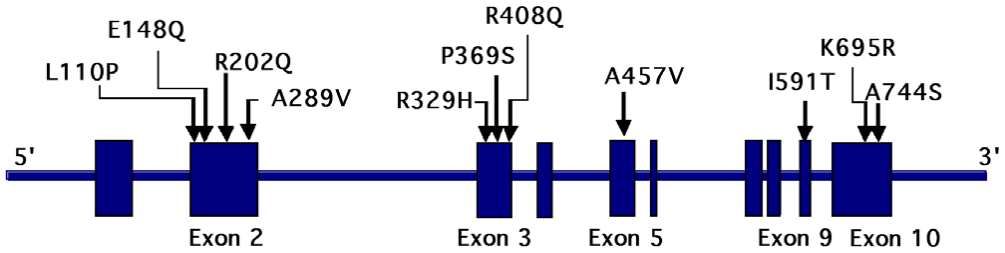
Missense mutation detected in the MEFV gene in FMS patients. Part of the genomic organization of the *MEFV* gene and a map of the missense mutations in patients with Fibromyalgia Syndrome are illustrated.

Based on our previous finding that FMS patients and their family members had high plasma levels of several cytokines or chemokines compared to unrelated controls [18], we hypothesized that the patients had inherited an inflammatory gene. Although a large number of inflammatory genes have been identified in the human genome, the *MEFV* gene family stands out as one of the founding members of a subgroup containing the PYD domain that has been linked to the so called inflammasome. We therefore began our search for a candidate gene in FMS by analyzing the *MEFV* gene in one hundred probands with FMS and their parents, all meeting the ACR criteria for FMS, were recruited into the study, along with 2 affected siblings. The clinical characteristics of the patient population were previously published [18]. The *MEFV* gene was analyzed in the 100 trios by direct sequencing, a total of 2.63 megabases of genomic sequence. One common and 10 rare missense variants were identified (**Table 1** **,** **Figure 1**). Seven probands (FMS45, 127, 254, 316, 495, 501, 549) had one heterozygous rare mutation; five probands (FMS52, 248, 321, 411, 435) had two rare variants; and three probands and one affected sibling had three or more rare variants (FMS51, 53, 512, 540). The family pedigree for FMS53 revealed transmission of two different rare variant haplotypes for two siblings (**Figure S1**). In total, 15 out of 100 probands (and one affected sibling) carried one or more rare variants (15%) in 10 distinct rare haplotypes. In addition to these rare missense variants, the common missense polymorphism, R202Q, was identified in 40 probands (40/100 = 40%). The allele frequency of R202Q overall was 23% (46/200), compared with 29.6% reported in dbSNP controls.

**Table 1:** MEFV mutations identified in the trios.

SNP ID	Variants	Exon	Associated phenotype^1^	Allele frequency (%)		Conservation
				FMS	dbSNP^2^	
rs11466018	L110P	2	Unknown	1.5	2.1	chimpanzee, monkey
rs3743930	E148Q	2	FMF atypical; FMF with criteria	3.5	2.1	mouse, rat, chimpanzee, monkey, rabbit, cow
rs224222	R202Q	2	Unknown	22.5	29.6	chimpanzee, rabbit, cow
n/a	A289V	2	FMF with criteria	0.5	n/a	chimpanzee, monkey
n/a	R329H	3	Unknown	0.5	n/a	chimpanzee, monkey, rabbit
rs11466023	P369S	3	Unknown	3.5	4.4	mouse, rat, chimpanzee, monkey, rabbit, cow
rs11466024	R408Q	3	Unknown	3.5	2.4	mouse, rat, chimpanzee, monkey, rabbit, cow
n/a	A457V	5	Unknown	0.5	n/a	chimpanzee, monkey
rs11466045	I591T	9	FMF with criteria	1.1	1.3	chimpanzee, monkey, rabbit
n/a	K695R	10	FMF with criteria	0.5	n/a	chimpanzee, monkey, rabbit
rs61732874	A744S	10	FMF with criteria	0.5	n/a	chimpanzee

1 Infevers Database: http://fmf.igh.cnrs.fr/ISSAID/infevers/; a diagnosis of FMF requires compound heterozygotes.

2 NCBI SNP database: www.ncbi.nlm.nih.gov/projects/SNP/

Given that 15% of the probands had one or more rare variants (**Table 1**), and our intention was to determine if the rare variants were inherited in a biased manner, it was clear that the analysis of multiple subgroups would be underpowered. Based on the fact that multiple rare *MEFV* variants were capable of causing FMF if present on both chromosomes, we decided to test the transmission of rare variants from parents to offspring **collectively**. We hypothesized that FMS, unlike FMF, would require any of the rare variants on a single chromosome. Thus the rare variants **collectively** would create a risk for FMS but would require a further event such as an environmental factor to trigger disease. **Table 2** lists the 22 independent events in which a rare allele was available for transmission from a parent to an affected proband or sibling. There are multiple lines for some families, with each line showing which haplotype was transmitted from a **heterozygous** parent to an affected child. As stated above, we tested the rare variants **collectively** for preferential transmission using the exact binomial version of the transmission disequilibrium test (TDT), as described in the Discussion section. In 17 of these 22 events (independent under the null hypothesis), the rare allele was transmitted to the affected offspring (p = 0.0085). The common allele R202Q was excluded from the analysis because it is frequently found in the general population [44] and does not qualify as rare. As expected, the same analysis for the common missense change, R202Q, showed no transmission bias to affected progeny (**Table S2**). Thus, we obtained evidence that rare alleles, encoding missense changes for the *MEFV* gene, were associated with FMS. The lack of biased transmission of the common allele from parents to probands suggested that R202Q does not correlate with risk of FMS and serves as an internal control for transmission of SNPs within this gene. It should be noted that in transmission analysis, the parents serve as the “controls” in that transmission of heterozygous rare alleles from parent to offspring should occur at a frequency of 0.5 by chance and >0.5 if associated with FMS.

**Table 2:** Transmission of rare variants for FMS trios.^1,2^

haplotypes	proband		mother		father		allele	
	ID#	genotype	ID#	genotype	ID#	genotype	ut (C)	t (B)
E148Q	FMS79	wt	FMS80	wt	FMS81	het	1	0
	FMS203	wt	FMS204	wt	FMS205	het	1	0
	FMS316	het	FMS317	het	FMS318	wt	0	1
L110P/E148Q	FMS52	het	FMS54	wt	FMS55	het	0	1
	FMS53	het	FMS54	wt	FMS55	het	0	1
	FMS248	het	FMS247	het	FMS249	wt	0	1
R329H	FMS45	het	FMS47	wt	FMS46	het	0	1
P369S/R408Q	FMS52	wt	FMS54	het	FMS55	wt	1	0
	FMS53	het	FMS54	het	FMS55	wt	0	1
	FMS321	het	FMS322	het	FMS323	wt	0	1
	FMS340	wt	FMS339	wt	FMS365	het	1	0
	FMS366	wt	FMS339	wt	FMS365	het	1	0
	FMS411	het	FMS464	wt	FMS463	het	0	1
	FMS435	het	FMS564	wt	FMS508	het	0	1
E148Q/P369S/R408Q	FMS51	het	FMS49	wt	FMS50	het	0	1
	FMS512	het	FMS511	wt	FMS510	het	0	1
E148Q/P369S/R408Q/A457V	FMS540	het	FMS539	wt	FMS538	het	0	1
A289V	FMS501	het	FMS503	wt	FMS502	het	0	1
I591T	FMS495	het	FMS497	wt	FMS496	het	0	1
	FMS549	het	FMS548	het	FMS547	wt	0	1
K695R	FMS254	het	FMS257	het	FMS258	wt	0	1
A744S	FMS127	het	FMS126	wt	FMS130	het	0	1
**Total**							5	17

1 Abbreviations are: wt-wildtype; het: heterozygote.

2 Probands FMS52 and FMS53 are sisters that both inherited one set of rare alleles from the father, while one of the sisters inherited rare variants from her mother (see Supplementary Fig S1.)

**Figure 2:**
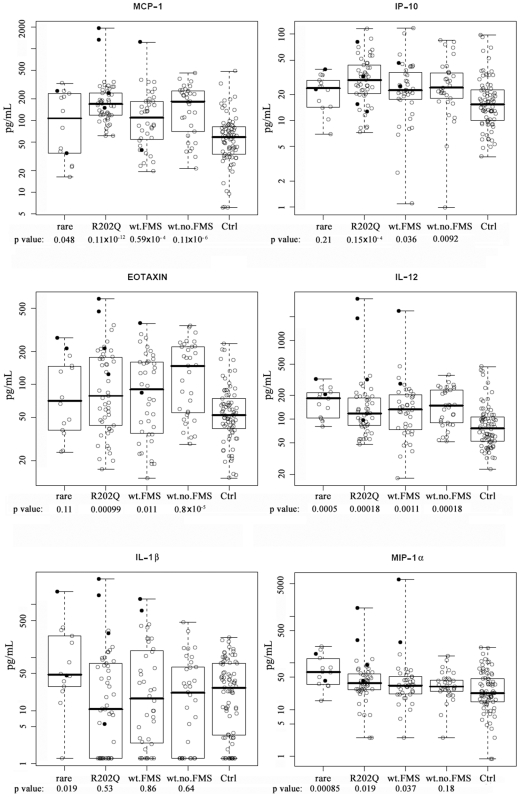
Plasma chemokine/cytokine levels by *MEFV* genotypes in FMS patients and family members, and in unrelated controls. Ctrl: plasma levels (pg/mL) for unrelated controls (n = 77) of unknown genotype. Wt no FMS: unaffected parents without variant alleles (n = 35). Wt FMS: FMS probands (n = 37) with wild type (non-variant) *MEFV* gene. R202Q: FMS probands and family members with R202Q genotype (n = 49). Rare: FMS patients with a rare variant of the *MEFV* gene (n = 14). P values are shown below each group. Boxplots indicate the median (heavy bar), central 50% of data (box) and range of observations (whiskers). P-values are from two-sided t-tests contrasting each group with the control group, using pooled variance, a logarithmic scale, and without any adjustment for multiple comparisons. In addition, subjects with the rare *MEFV* alleles compared to wt FMS patients had elevated levels of MIP-1α (p = 0.019); subjects with R202Q polymorphism compared to wt FMS patients had elevated levels of MCP-1 (p = 0.004).

Since we had previously shown that both patients and their parents had high plasma levels of MCP-1, IP-10 and eotaxin compared to unrelated controls [18], we decided to re-analyze these data to test if chemokine and/or cytokine plasma levels were correlated with the rare *MEFV* genotypes. As shown in **Figure 2**, MCP-1, IP-10 and eotaxin plasma levels were elevated, on average, for all FMS patients vs. unrelated controls, *except* those with the rare alleles for the MEFV gene, who were empirically intermediate, but cannot be statistically distinguished from either controls or other FMS patients. However, levels of IL-1β, the chief cytokine associated with functional analysis of the pyrin protein (protein product of the *MEFV* gene), were high in FMS patients with the rare *MEFV* alleles, but not in unrelated controls or FMS patients with wild type *MEFV*. Plasma levels of MIP-1α, a chemokine involved in monocyte migration and activation [45], were elevated in subjects with the rare *MEFV* alleles compared to other FMS patients (p = 0.019, legend to **Figure 2**), who, along with their relatives, had elevated levels compared to unrelated controls. MCP-1, a chemokine with similar function, showed no such distinction of the individuals with rare alleles, but those with the R202Q polymorphism were strikingly elevated (p = 0.004, legend **Figure 2**) compared to FMS patients generally, and all FMS patient and family groups were elevated relative to unrelated controls. FMS patients and family members without rare variants differed from control subjects with regard to both T_H_1 (IFNγ) and T_H_2 (IL-5 and IL-13) cytokine levels (**Figure S2**). Those with rare alleles were similar to other FMS patients and family with regard to IL-5 levels, but similar to control subjects and distinct from other FMS patients without rare variants (p = 0.003) with regard to IFNγ, and indistinguishable from either group with regard to IL-13. However, we also noted a significant elevation for IL-12 plasma levels for all groups compared to unrelated controls (**Figure 2**). In spite of the expected relationship between IL-12 and IFNγ [46] in T_H_1-like diseases, there was no elevation in IFNγ levels (**Figure S2**), arguing against a T_H_1 involvement in FMS.

## Discussion

### FMF-Related *MEFV* Variants

Since we have proposed that FMS has an underlying basis in inflammation [18], we began our search for a candidate gene with the well-studied *MEFV* gene associated with FMF. In addition, we examined *NLRP3* (C1AS1 showed a single missense mutation in its exons) and 3 other genes associated with the inflammasome (ASC1, ASC2, and CD2BP1 exhibited no missense mutations in their exons). Since our cohort utilized trios, it was possible to measure transmission of haplotypes directly from parents to probands without the need for extensive matching of age, ethnicity, and other factors that might confound other designs. Thus, the parents serve as “controls” for the FMS affecteds. Among the genes analyzed (data not shown) only the *MEFV* gene displayed a significant result in terms of rare alleles that were transmitted from parents to probands. Of interest, these other genes collectively demonstrated only a few rare missense mutations.

Our data herein provide strong evidence for the association between the *MEFV* structural gene variants and a subset (15%) of FMS patients. Of the 10 rare missense variants we identified, five are FMF-related mutations (E148Q, A289V, I591T, K695R, A744S; INFEVERS Database: http://fmf.igh.cnrs.fr/ISSAID/infevers/.) E148Q is one of the most common mutations in FMF patients. It has been proposed that E148Q (together with another FMF allele) causes a milder disease, although some reports indicated E148Q should be classified as a polymorphism, rather than a disease-causing mutation, since it occurs at a higher frequency in the general population (**Table 1**). Interestingly, we found three FMS patients carrying three different mutations, E148Q/P369S/R408Q, which were reported previously in an FMF patient, all carried in cis on the same allele/haplotype [47]. No founder mutations (M680V, M694I, M694V and V726A) of the *MEFV* gene were identified in our study. These results support our diagnosis of FMS in this cohort, rather than variant FMF. Importantly, only a subset of the patient cohort carry the rare *MEFV* variants, leaving open the question of which genes are associated with risk in the remaining 85% of the patient cohort.

### Significance of the Common Mutation, R202Q

R202Q has previously been reported as a common polymorphism [44]. There is some evidence suggesting that R202Q homozygosity increases risk of FMF [48]. Homozygosity of R202Q has been detected in four of 26 FMF patients and none of 60 healthy individuals (p = 0.007, [49]). The lack of this homozygous alteration in 60 healthy individuals and nine patients suffering from other inflammatory diseases raises the possibility of a cooperative role for R202Q/R202Q in FMF. Although heterozygous R202Q is common among healthy individuals [49], its homozygosity in FMF patients may reflect a dosage-dependent deleterious effect that might require other genetic and environmental elements for expression. We identified six homozygous R202Q individuals in 100 FMS probands (6%). Analysis of a larger cohort and functional studies of the R2022Q variant pyrin protein may assist in understanding the role of this variant in both FMS and FMF.

### Statistical Methodology

Collective testing of haplotypes carrying rare mutations is motivated by the variety of rare variants that contribute to FMF as compound heterozgotes and the variety of rare variants acting in a dominant mode at the cryopyrin gene. The collective testing of rare alleles at candidate genes is an increasingly common strategy in genetic epidemiology [50]. Pritchard [51] argues that rare alleles are, collectively, major contributors to common disorders; and Kryukov et al [43] assert that rarity of an allele is, by itself, an indicator of likely deleterious effect. Schork et al. [52] state that statistical testing of collections of rare variants, as a group, is a new but “crucial” development in genetic epidemiology.

Because we were interested in testing for dominant genetic effects (only one affected child was a compound heterozygote) and because no parents were compound heterozygotes, we were able to apply a particularly simple test. There were 22 transmission events in which a heterozygous parent could pass a chromosome with or without rare variants to their child. If the *MEFV* variants were neutral (the null hypothesis), either chromosome would be equally likely to be transmitted; furthermore, these events are stochastically independent. If rare variants increase the risk of FMS, then the fact that affected offspring are sampled will increase the probability of finding rare variants. We can simply count the number of transmissions and calculate the significance probability (p-value) from the binomial distribution, with event probability 0.5, and n = 22. This is a test of transmission, but it differs from the commonly used transmission/disequilibrium test (TDT) in that it tests rare variants collectively, is one-sided, and only involves only one mating type. The more commonly employed approximate test refers (b−c)^2^/(b+c) to a chi-square distribution, where b and c are the respective counts of transmitted and untransmitted candidate alleles from heterozygous parents; but, as Spielman et al. [53] noted when proposing this test, “An exact binomial test can be used, if desired…” Spielman et al. go on to describe an application to multiple markers in the same manner that we use here; but, as they were concerned with markers, they imagined collecting alleles based on prior evidence of association rather than collecting uncommon missense variants. The major implications of using the TDT statistic to test a collection of uncommon missense sequences instead of markers are that (1) the test becomes one-sided (or there is no basis for collecting multiple variants); (2) the concern about segregation distortion is greatly reduced; and (3) the various marker-oriented characterizations of the TDT as a test of linkage in the presence of association become meaningless, as there is no marker.

Slager, et al, [54] have shown that the TDT has a dramatic loss of power when there is allelic heterogeneity. Our one-sample binomial test handles allelic heterogeneity by simply counting all of the rare alleles, and maintains the advantages of a family-based test of linkage and association, essentially using untransmitted alleles as controls, which provides robustness against spurious conclusions induced by hidden genetic structure, such as stratification or admixture. Thus, there is no need to obtain and study age- and ethnicity-matched controls.

There is a growing discussion among human geneticists that collections of rare alleles at pathogenetic loci will account for a significant proportion of genetically-based human disorders [52]. Thus, we feel confident that the analysis presented here is the most appropriate test for studying the possible association of FMS with the *MEFV* gene at this early stage of analysis. Our data suggest that transmission of rare *MEFV* alleles constitutes a strong risk factor, but only for a subset of FMS patients.

### Correlative Studies

The correlation of rare *MEFV* alleles with elevated chemokine/cytokine plasma levels is especially intriguing because functional studies on the pyrin protein would predict disregulation of IL-1β secretion in patients with chronic inflammation. In our previous analysis on a subset of patients and parents with FMS, we found elevation of MCP-1 and eotaxin plasma levels [18], but the standard deviation on IL-1β levels was large in both controls and FMS patients, providing no significant difference between the two groups. However, upon re-analysis of the data comparing FMS patients with rare *MEFV* alleles to controls, IL-1β was significantly elevated (p = 0.019). This is the first demonstration of such a correlation and merits further study in both FMS and FMF patients. What remains unexplained is the cause of the high MCP-1 and eotaxin levels in FMS patients, regardless of their genotype. This suggests that there is an environmental component to the disease, most likely underlying chronic inflammation, since many inflammatory stimuli will cause the production of MCP-1, which, in turn, will activate monocytes. While no evidence of monocytic infiltrates into skeletal muscle has been found in FMS patients, there is evidence of skeletal muscle damage [22] that may be caused by high systemic levels of MCP-1. MCP-1 has been shown to up-regulate IL-1β expression in monocytes [55]. The specific interaction of MCP-1 with skeletal muscle has not been studied in detail, but we have shown that MCP-1 stimulates the release of IL-1β, IP-10 and eotaxin from myoblasts [18]. In addition, eotaxin is the natural antagonist for the CCR2 receptor, the main receptor for MCP-1 [56]. Thus, high levels of eotaxin may reflect an attempt by skeletal muscle (or other cells) to counteract the effects of MCP-1. Finally, MCP-1 has a direct effect on sensory neurons, causing pain [57], and reduces glucose uptake in skeletal muscles [58], possibly subjecting skeletal muscles to fatigue. What remains to be explored is the complex connection between the effect of mutations in inflammation-regulating genes, such as *MEFV*, and environmental effects that sustain the production of harmful chemokines and cytokines, such as MCP-1, eotaxin, and IL-1β.

The lack of the usual wide variation in plasma MCP-1 and IP-10 levels in FMS patients with the common R202Q allele may indicate that this variant has a functional connection to the production of these two cytokines, rather than IL-1β, as seen in the patients with rare *MEFV* alleles. It is also noteworthy that patients with the common polymorphism have depressed levels of IFNγ and IL-13, the former demonstrating a lack of T_H_1-like disease (where high IFNγ and low IL-13 are expected), and the latter arguing against T_H_2-like disease, such as asthma (where high IL-4 and IL-13 are expected). Taken together, these data argue against a T_H_1 or T_H_2 origin for the disease in the 15% of patients with rare *MEFV* alleles and the 40% of probands with the common variant. (In this regard, it is interesting that IL-1β is associated with innate immunity.) Thus, polymorphisms in the *MEFV* gene may predispose up to 55% of probands to an immunological imbalance in cytokine/chemokine levels.

Finally, we have accounted for a possible disease susceptibility gene in only a subset of FMS. Given the large number of NLR genes and their growing number of disease associations, it is likely that more NLR gene variants will be found as risk factors for FMS, and that specific chronic infections will be identified that trigger FMS via their interactions with these genes. Therefore, we are hopeful that the etiology of FMS will emerge along with its correlation to genetic risk factors. Importantly, the studies presented here are correlative and do not provide a mechanistic explanation for FMS. Studies are now underway to determine if the rare variants identified confer a phenotype to transfected cell lines.

## Methods

### Subjects

The study was approved by the Institutional Review Board of City of Hope National Medical Center (IRB 04186). Patients with fibromyalgia recruited into the study contacted their first-degree relatives, who were subsequently also recruited into the study. Written informed consent was obtained from all participants in this study. Probands and affected parents were diagnosed with FMS fulfilling ACR criteria [3]. Patients with the autoimmune diseases rheumatoid arthritis (RA) and systemic lupus erythematosus (SLE) were excluded from the study; other clinical characteristics of the patient population have been previously described [18]. Unrelated control subjects, as previously described [18] were used for comparison of cytokine/chemokine levels, but the genetic analysis used the transmission disequilibrium test, which avoids the need for unaffected control subjects.

### PCR and Sequencing

DNA was isolated from peripheral leukocytes or saliva, using the QIAamp DNA Blood Mini Kit (Qiagen) and Oragene DNA Self Collection Kits (DNA Genotek) according to the manufacturers' instructions. All coding exons and splice junctions (except exon 2) of the *MEFV* gene were amplified by PCR in a total volume of 20 µl with 10 mM Tris-HCl, pH 8.3, 50 mM KCl, 1.5 mM MgCl_2_, 200 µM of each deoxyribonucleoside triphosphate, and 0.2 µM of primers. 1 U of Ampli-Taq Gold (Roche) and 20 ng of genomic DNA were added. Exon 2 was amplified with the GC-RICH PCR System (Roche). PCR reactions were performed on the GeneAmp PCR System 9700 (Applied Biosystems) with denaturation at 94°C for 10 min, and then denaturation at 94°C for 15 sec, annealing at 60°C for 30 sec, and elongation at 72°C for 1 min for a total of 35 cycles and a final elongation for 10 min at 72°C. The amplicons were purified by ExoSAP-IT and sequenced with the ABI PRISM 3730 (Applied Biosystems). The sequences of PCR primers are listed in **Table S1**. Genomic and amino acid sequences for *MEFV* were collected from Ensemble: ENST00000219596, ENSG00000103313.

### Statistical Analysis

The statistical considerations are described in detail in the Discussion section.

### Measurement of Cytokines and Chemokines

The plasma level of cytokines and chemokines of a portion of the cohort described here were previously reported [18]. The levels were compared in unrelated controls and probands and family members with wild type *MEFV* or variant *MEFV* alleles. Statistical analysis was performed by a two sided, unpaired student's t test.

## Supporting Information

Figure S1 Pedigree for family 13 that transmitted multiple rare alleles to two different offspring. Filled circles are females with FMS, open square, unaffected father.
(0.54 MB TIF)

Figure S2 Cytokines that inversely correlate with rare alleles in FMS patients and their families. Ctrl: plasma levels (pg/mL) for unrelated controls (n = 77), unknown genotype. Wt no FMS: parents with wild type alleles and no FMS (n = 35). Wt FMS: FMS probands (n = 37) with wild type *MEFV* gene. R202Q: FMS probands and family members with R202Q genotype (n = 49). Rare: FMS patients with a rare variant of the *MEFV* gene (n = 14). P values are shown below each group. Boxplots indicate the median (heavy bar), central 50% of data (box) and range of observations (wiskers). P-values are from two-sided t-tests contrasting each group with the control group, using pooled variance, a logarithmic scale, and without any adjustment for multiple comparisons.
(1.00 MB TIF)

Table S1 Primers for the *MEFV* gene.
(0.04 MB DOC)

Table S2 Genotype of R202Q in trios.
(0.07 MB DOC)
