# Supplementary figures and images for: Missense Mutations in the MEFV Gene Are Associated with Fibromyalgia Syndrome and Correlate with Elevated IL-1β Plasma Levels
Source: PLoS One. 2009 Dec 30;4(12):e8480. doi: 10.1371/journal.pone.0008480 (PMC2794536; doi:10.1371/journal.pone.0008480)

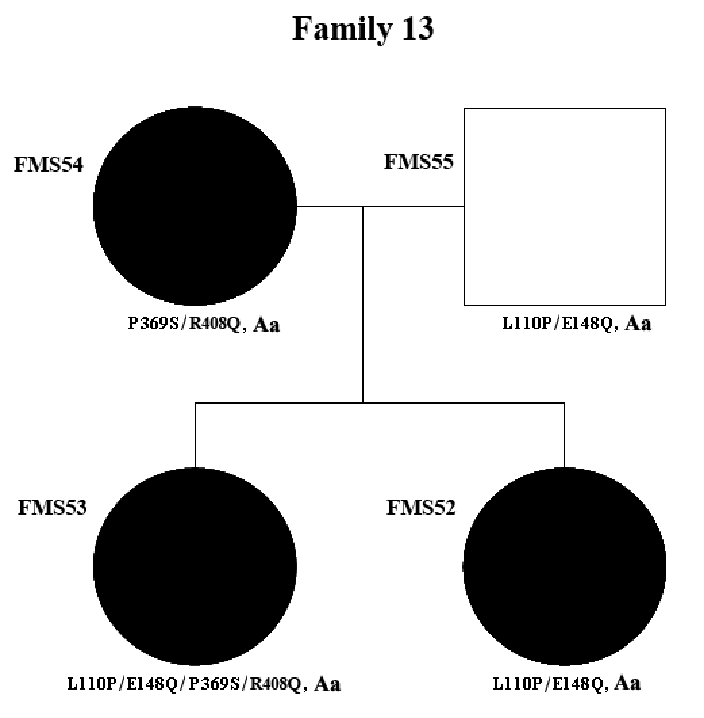

Supplement: Figure S1 — Pedigree for family 13 that transmitted multiple rare alleles to two different offspring. Filled circles are females with FMS, open square, unaffected father. (0.54 MB TIF) [file pone.0008480.s001.tif]

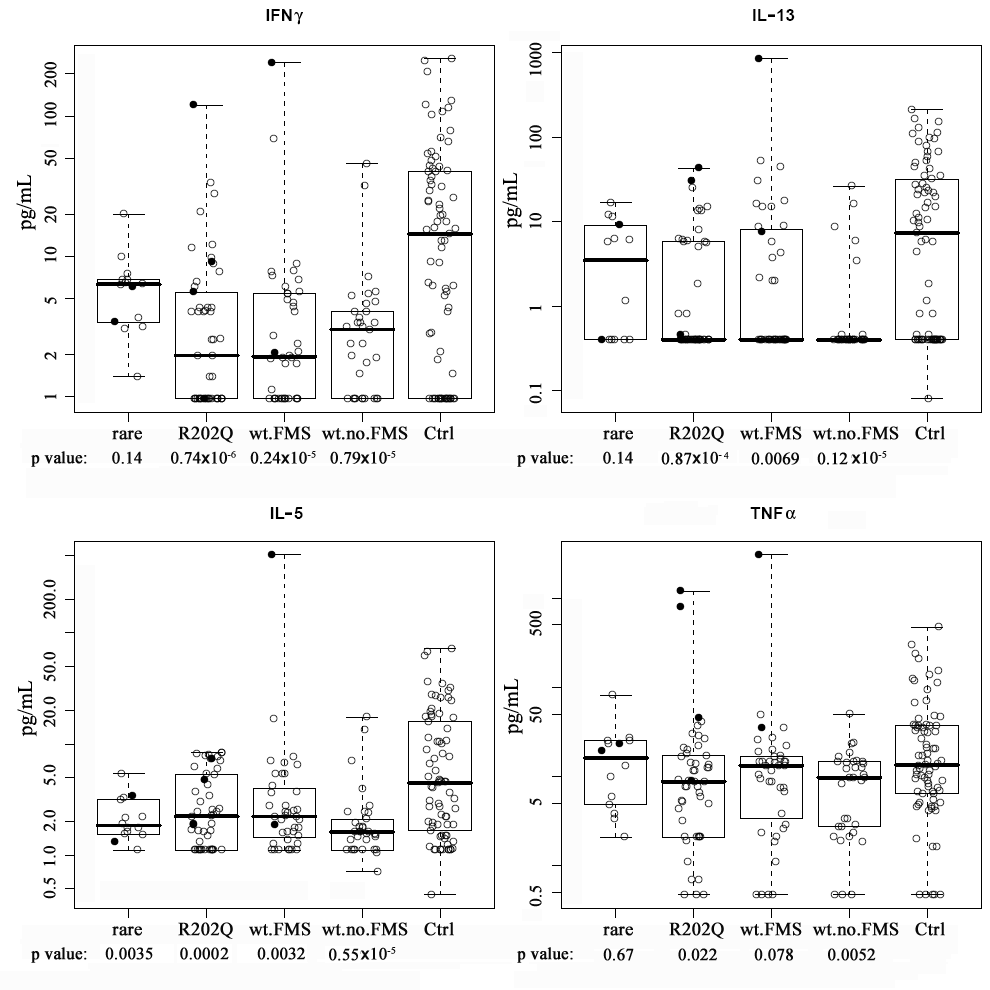

Supplement: Figure S2 — Cytokines that inversely correlate with rare alleles in FMS patients and their families. Ctrl: plasma levels (pg/mL) for unrelated controls (n = 77), unknown genotype. Wt no FMS: parents with wild type alleles and no FMS (n = 35). Wt FMS: FMS probands (n = 37) with wild type MEFV gene. R202Q: FMS probands and family members with R202Q genotype (n = 49). Rare: FMS patients with a rare variant of the MEFV gene (n = 14). P values are shown below each group. Boxplots indicate the median (heavy bar), central 50% of data (box) and range of observations (wiskers). P-values are from two-sided t-tests contrasting each group with the control group, using pooled variance, a logarithmic scale, and without any adjustment for multiple comparisons. (1.00 MB TIF) [file pone.0008480.s002.tif]
