# Supplementary material for: Missense Mutations in the MEFV Gene Are Associated with Fibromyalgia Syndrome and Correlate with Elevated IL-1β Plasma Levels
Source: PLoS One. 2009 Dec 30;4(12):e8480. doi: 10.1371/journal.pone.0008480 (PMC2794536; doi:10.1371/journal.pone.0008480)
Supplement: Table S1 — Primers for the MEFV gene. (0.04 MB DOC) [file pone.0008480.s003.doc]

**Table S1. Primers for the MEFV gene.**

| **Exon** | **size (bp)** | **name** | **PCR Primers** | **Size (bp)** |
| --- | --- | --- | --- | --- |
| 1 | 277 | E1D2 | cacaacctgccttttcttgc | 420 |
|  |  | E1U2 | caaagcagccagcactcag |  |
| 2 | 633 | E2Da | tctcctctgccctgaatct | 587 |
|  |  | E2U1 | gagtcaggagaatttctgga |  |
| 2 | 633 | E2D2 | ctgagcaaacgcagagaga | 517 |
|  |  | E2U2 | ttacaggcatgagctatcgt |  |
| 3 | 350 | E3D | gtgctttgtgatacctctgt | 488 |
|  |  | E3U | aatgaagtaaggcccagtgt |  |
| 4 | 96 | E4D | agttggcaccagctaaagat | 251 |
|  |  | E4U | tgtgaaccacagcagaatct |  |
| 5 | 231 | E5D | tcctggacatccacgtcc | 393 |
|  |  | E5U | tcacccacttgttccagca |  |
| 6 | 23 | E6D2 | tgctccacttccactgaca | 295 |
|  |  | E6U2 | gaggagtctggaatcacaga |  |
| 7 & 8 | 116 & 33 | E7D | atgtagttcatttccagctca | 483 |
|  |  | E8U | aagtcaacagcacaagggaa |  |
| 9 | 33 | E9D | ttccttgttgtcaggacagt | 200 |
|  |  | E9U | acagggtagttcttctggaa |  |
| 10 | 553 | E10D1 | ttccagaagaactaccctgt | 760 |
|  |  | E10U2 | tgcatttcccatagcagcta |  |
