# Supplementary material for: Missense Mutations in the MEFV Gene Are Associated with Fibromyalgia Syndrome and Correlate with Elevated IL-1β Plasma Levels
Source: PLoS One. 2009 Dec 30;4(12):e8480. doi: 10.1371/journal.pone.0008480 (PMC2794536; doi:10.1371/journal.pone.0008480)
Supplement: Table S2 — Genotype of R202Q in trios. (0.07 MB DOC) [file pone.0008480.s004.doc]

**Table S2. Genotype of R202Q in trios^1^.**

| **Trios #** | **proband** | **mother** | **father** | **ut(c)** | **t(b)** |
| --- | --- | --- | --- | --- | --- |
| 5 | het | het | het | 1 | 1 |
| 14 | het | wt | het | 0 | 1 |
| 15 | het | het | het | 1 | 1 |
| 16 | het | wt | het | 0 | 1 |
| 17 | het | wt | het | 0 | 1 |
| 18 | het | het | wt | 0 | 1 |
| 22 | wt | het | wt | 1 | 0 |
| 23 | het | het | wt | 0 | 1 |
| 25 | wt | het | het | 2 | 0 |
| 28 | het | het | wt | 0 | 1 |
| 32 | wt | wt | het | 1 | 0 |
| 33 | het | wt | het | 0 | 1 |
| 35 | homo | het | het | 0 | 2 |
| 37 | het | het | wt | 0 | 1 |
| 38 | het | het | wt | 0 | 1 |
| 40 | wt | het | wt | 1 | 0 |
| 42 | het | wt | het | 0 | 1 |
| 43 | wt | het | wt | 1 | 0 |
| 46 | wt | het | het | 2 | 0 |
| 49 | het | het | het | 1 | 1 |
| 52 | het | het | wt | 0 | 1 |
| 54 | het | het | wt | 0 | 1 |
| 65 | wt | het | wt | 1 | 0 |
| 57 | wt | wt | het | 1 | 0 |
| 59 | het | het | wt | 0 | 1 |
| 62 | wt | het | het | 2 | 0 |
| 64 | het | het | wt | 0 | 1 |
| 66 | wt | wt | het | 1 | 0 |
| 70 | wt | het | wt | 1 | 0 |
| 71 | wt | het | het | 2 | 0 |
| 72 | wt | het | wt | 1 | 0 |
| 75 | het | wt | het | 0 | 1 |
| 77 | het | het | het | 1 | 1 |
| 78 | wt | het | het | 2 | 0 |
| 84 | wt | wt | het | 1 | 0 |
| 85 | het | het | het | 1 | 1 |
| 86 | wt | wt | het | 1 | 0 |
| 90 | het | het | wt | 0 | 1 |
| 91 | het | het | wt | 0 | 1 |
| 94 | wt | het | het | 2 | 0 |
| 95 | homo | het | het | 0 | 2 |
| 96 | het | het | het | 1 | 1 |
| 97 | wt | wt | het | 1 | 0 |
| 98 | wt | het | het | 2 | 0 |
| 100 | homo | het | het | 0 | 2 |
| Total |  |  |  | 32 | 29 |

1. wt-wildtype; het: heterozygote; homo: homozygote; ut(c): untransmitted allele; t(b): transmitted allele.
